# Metabolic phenotyping in people living with obesity: Implications for dietary prevention

**DOI:** 10.1007/s11154-023-09830-4

**Published:** 2023-08-15

**Authors:** Ellen E. Blaak, Gijs H. Goossens

**Affiliations:** Department of Human Biology, NUTRIM School of Nutrition and Translational Research in Metabolism, Maastricht University Medical Center+, P.O. Box 616, 6200 MD Maastricht, The Netherlands

**Keywords:** Metabotype, Tissue-specific insulin resistance, Precision nutrition, Metabolically (un)healthy obesity, Cardiometabolic risk

## Abstract

Given the increasing number of people living with obesity and related chronic metabolic disease, precision nutrition approaches are required to increase the effectiveness of prevention strategies. This review addresses these approaches in different metabolic phenotypes (metabotypes) in obesity. Although obesity is typically associated with an increased cardiometabolic disease risk, some people with obesity are relatively protected against the detrimental effects of excess adiposity on cardiometabolic health, also referred to as ‘metabolically healthy obesity’ (MHO). Underlying mechanisms, the extent to which MHO is a transient state as well as lifestyle strategies to counteract the transition from MHO to metabolically unhealthy obesity (MUO) are discussed. Based on the limited resources that are available for dietary lifestyle interventions, it may be reasonable to prioritize interventions for people with MUO, since targeting high-risk patients for specific nutritional, lifestyle or weight-loss strategies may enhance the cost-effectiveness of these interventions. Additionally, the concept of tissue insulin resistant (IR) metabotypes is discussed, representing distinct etiologies towards type 2 diabetes (T2D) as well as cardiovascular disease (CVD). Recent evidence indicates that these tissue IR metabotypes, already present in individuals with obesity with a normal glucose homeostasis, respond differentially to diet. Modulation of dietary macronutrient composition according to these metabotypes may considerably improve cardiometabolic health benefits. Thus, nutritional or lifestyle intervention may improve cardiometabolic health, even with only minor or no weight loss, which stresses the importance of focusing on a healthy lifestyle and not on weight loss only. Targeting different metabotypes towards T2D and cardiometabolic diseases may lead to more effective lifestyle prevention and treatment strategies. Age and sex-related differences in tissue metabotypes and related microbial composition and functionality (fermentation), as important drivers and/or mediators of dietary intervention response, have to be taken into account. For the implementation of these approaches, more prospective trials are required to provide the knowledge base for precision nutrition in the prevention of chronic metabolic diseases.

## Introduction

The prevalence of obesity, insulin resistance (IR) and related chronic metabolic diseases such as type 2 diabetes (T2D), cardiovascular diseases (CVD) and mental diseases has grown dramatically over the past decades, with far reaching consequences for individuals, society and economy [1, 2].

A healthy diet may improve cardiometabolic health, even in the presence of minor weight loss [3]. Nevertheless, although preventive strategies such as lifestyle interventions have improved over time, long-term maintenance and adherence to a healthy lifestyle remains poor [4]. It is becoming progressively evident that the concept of a universal dietary solution does not apply when considering lifestyle or dietary strategies for enhancing health, as a substantial subset of individuals does not respond to dietary interventions. It is widely recognized that only about 40% of the study population exhibit a beneficial metabolic response to generic dietary interventions, wherein the responsiveness is closely linked to distinct metabolic phenotypes, so-called metabotypes [5–8]. The usage of machine learning algorithms to improve blood glucose control has been proven successful [9–11]. In a retrospective analysis of the Tubingen Lifestyle Intervention Program, a high-risk metabotype was identified, characterized by beta-cell dysfunction and/or insulin-resistant (IR) nonalcoholic fatty liver disease with higher probability of long-term non-response to lifestyle intervention [12]. In the latter group, intensification of lifestyle intervention showed a higher improvement in glucose tolerance [13]. Additionally, we recently provided, for the first time, the proof-of-concept that isocaloric dietary macronutrient modulation according to tissue IR metabotype may considerably further improve insulin sensitivity and cardiometabolic health [14]. Combined, these data indicate that a precision-based approach to improve cardiometabolic health seems promising and may increase intervention efficacy as well as adherence to intervention.

This review will discuss the different metabotypes in obesity and their relationship with the risk of developing cardiometabolic disease, and highlight underlying (tissue-specific) metabolic disturbances. Next, our focus lies in assessing the efficacy of a precision-based approach that targets specific (tissue) metabotypes to enhance the success of nutritional or lifestyle interventions in individuals who are living with overweight or obesity. Results from recent dietary intervention studies that used a precision nutrition or lifestyle approach targeting specific metabotypes to further improve intervention effectiveness in individuals living with overweight or obesity will be discussed. Finally, future perspectives and approaches will be addressed.

## Metabolically healthy versus unhealthy obesity metabotypes

Although obesity is typically related to metabolic dysfunction and an elevated cardiometabolic disease risk, expansion of adipose tissue does not always result in metabolic perturbations. There is a group of individuals with obesity that is relatively protected against the detrimental effects of excess adiposity on cardiometabolic health, also alluded to as ‘metabolically healthy obesity’ (MHO). Several lines of evidence have shown that the absolute amount of body fat is not the main factor determining the metabotype in people with obesity. For example, abdominal liposuction did not improve metabolic health, including IR, in humans [15]. Furthermore, despite an increase in fat mass, pharmacological activation of PPARγ using thiazolidinediones increased insulin sensitivity in humans [16]. Another condition showing that there fat mass is not directly associated with metabolic health is lipodystrophy. Adipose tissue deficiency in patients with (partial) lipodystrophy is accompanied by IR and an increased risk of T2D [17].

**Fig. 1:**
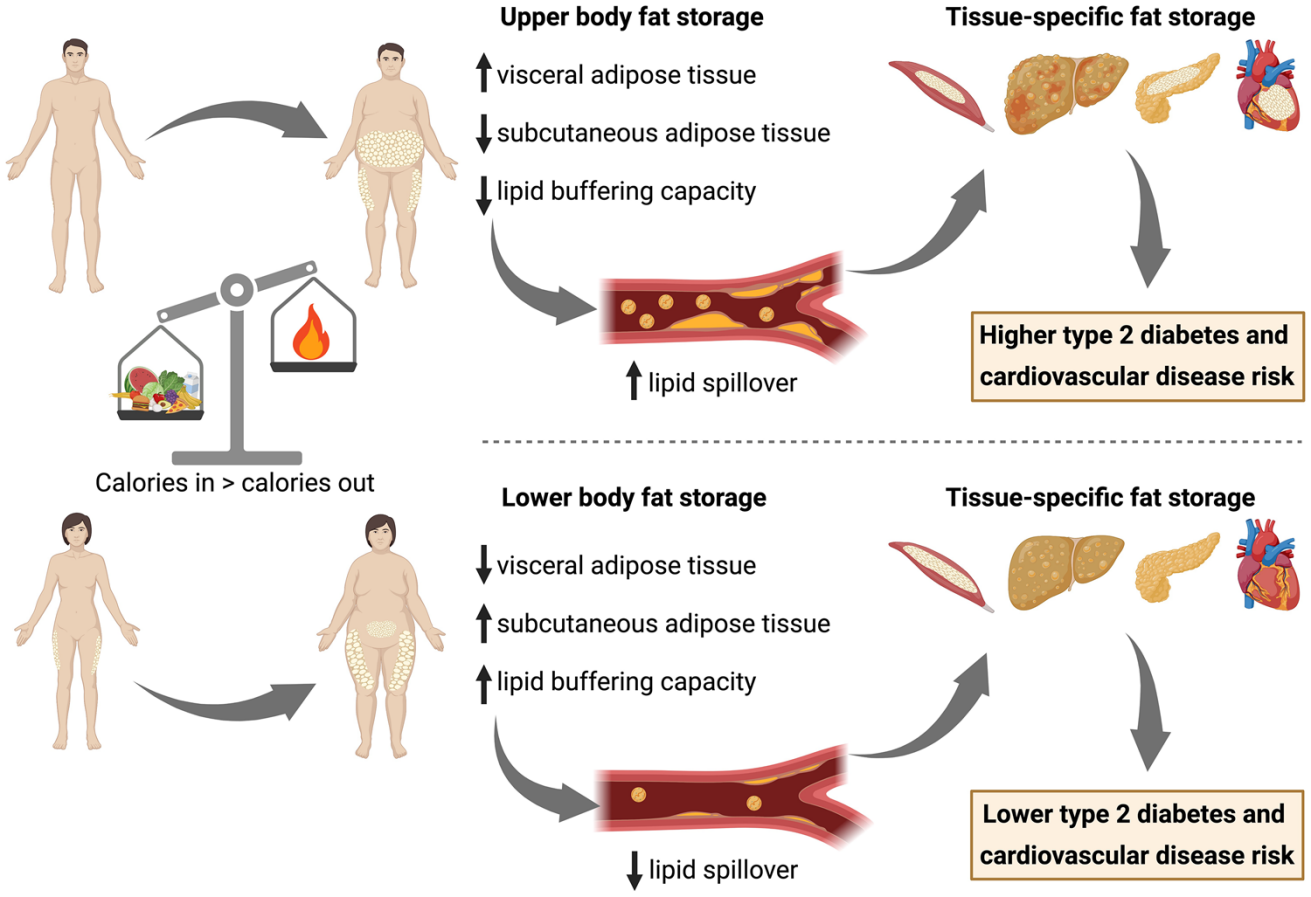
Body fat distribution is a key determinant of cardiometabolic disease risk. Subcutaneous adipose tissue buffers the daily influx of excess calories during a prolonged positive energy balance. Upper body fat storage in people with abdominal obesity (that is, predominant lipid storage in the abdominal region) is usually paralleled by more lipid spillover in the circulation, an increase in visceral adipose tissue and ectopic fat storage (that is, lipid accumulation in non-adipose such as skeletal muscle, liver, pancreas and heart), which instigates the development of insulin resistance and chronic cardiometabolic diseases such as type 2 diabetes and cardiovascular diseases. In contrast, predominant fat storage in the lower body as seen in people with lower body obesity limits lipid spillover in the circulation, since gluteo-femoral adipose tissue acts as a metabolic sink to buffer excess lipids. Consequently, less lipids will accumulate in visceral adipose tissue and certain non-adipose tissues, thereby reducing cardiometabolic disease risk. Premenopausal women living with obesity seem to be characterized by more lipid accumulation in skeletal muscle (smaller lipid droplets consisting of less saturated fatty acids) and similar or less lipid storage in the liver, with less detrimental effects on tissue-specific insulin sensitivity compared to men with obesity. Created with BioRender.com

The location where lipids are stored is a stronger risk factor for metabolic and cardiovascular diseases than excess adiposity per se. Abdominal obesity (fat accumulation in the upper body) is positively associated with the development of obesity-related comorbidities and all-cause mortality, while lower body obesity (fat accumulation in the gluteofemoral region) is related to a protective lipid and glucose profile, and a lower prevalence of cardiometabolic diseases after adjustment for total fat mass [18–21] (Fig. 1). Noteworthy, deep abdominal subcutaneous adipose tissue, which refers to the fat situated below Scarpa’s fascia, dividing the superficial and deep layers of abdominal subcutaneous fat, appears to increase disproportionately compared to the superficial fat as obesity progresses. This particular expansion tendency contributes to a higher susceptibility to cardiometabolic complications and chronic diseases in men, regardless of other measures of adiposity [22]. The risk associated with a certain body fat distribution pattern seems to be explained by strikingly distinct functional properties of different fat depots, as extensively reviewed elsewhere [23]. One important factor underlying the differential cardiometabolic disease risk between people with upper versus lower body obesity is that abdominal adipose tissue has a high lipid turnover or, in other words, is able to rapidly take up and store nutrients after meal intake and release fatty acids under fasting or exercise conditions. In contrast, lower body fat stores exhibit a diminished rate of lipid turnover and retain lipids that would otherwise be directed to non-adipose tissues [23]. Thus, lower body fat seems to act as a ‘metabolic sink’ that protects from ectopic fat deposition (i.e. fat deposition in skeletal muscle, the liver and visceral adipose tissue) and, consequently, insulin resistance and cardiometabolic complications [24–26] (Fig. 1). Although visceral fat mass is a key factor in cardiometabolic disease development compared with the amount of fat stored in other adipose tissue depots, there is evidence that a (relatively) low mass of lower body fat depots may independently predict cardiometabolic disease risk, suggesting that a reduced amount of lower body fat is as important as a high visceral fat mass regarding the risk of cardiometabolic disease development, at least in people with a normal weight [27].

In accordance with the importance of body fat distribution in cardiometabolic disease development, many studies have demonstrated that excess fat mass does not explain the distinct cardiometabolic profile between individuals with MHO and ‘metabolically healthy obesity’ (MUO). Rather, differences in the location where the excessive calories are stored seems to distinguish these obesity phenotypes. Indeed, several studies have shown that individuals with MHO have more subcutaneous adipose tissue (more gluteofemoral fat in women), less visceral fat mass, less fat accumulation in the liver and skeletal muscle, and less macrophage infiltration and inflammation in (visceral) adipose tissue than people with MUO, matched for age, sex, BMI and fat mass [27–39]. Of note, it seems that beside dietary intake the physical activity level and cardiorespiratory fitness are also important determinants of the metabotype in individuals with obesity [39, 40].

## Metabolically healthy obesity: a transient state?

To date, there is no universally accepted definition of MHO. Most studies that compared characteristics of people with MHO and MUO phenotypes and/or examined the association of MHO with the risk of developing chronic diseases or mortality defined MHO as having ≤ 2 components of the metabolic syndrome, although the homeostasis model assessment of insulin resistance (HOMA-IR) was used to define MHO in other studies [41]. This means that many study participants were likely ‘misclassified’ as having MHO, while these individuals might just had fewer cardiometabolic perturbations than those classified as having MUO. The latter is also important when interpreting the results of studies that reported the prevalences of MHO in different populations.

In addition to the criteria used to define MHO, the prevalences of MHO and MUO depend on several characteristics of the study populations such as BMI, age, sex, ethnicity and the absence/presence of other chronic diseases. These factors likely explain the large variability in reported MHO prevalence, ranging from ~5% (MHO defined as no metabolic syndrome component and normal HOMA-IR) to ~50% (MHO defined as no metabolic syndrome) [41]. It has been shown that MHO is more common in individuals with a BMI below 35 kg/m^2^ [42], younger adults [43–47], women [43–45], and people of European ancestry compared to those from South Asia, South America and Africa [45, 48].

The risk of developing cardiovascular diseases among individuals in various BMI categories is likely influenced by their metabolic health. An key question is whether and to what extent metabolic health in people with MHO deteriorates over time and how this impacts cardiometabolic disease risk. Several prospective cohort studies and meta-analyses have demonstrated that the majority of people with MHO has an increased risk of T2D and cardiovascular disease compared to healthy people with normal body weight [49–51]. A meta-analysis demonstrated that MHO was related to a higher risk of events when only studies with 10 or more years of follow-up were considered [51]. Longitudinal studies indicate that 30–50% of MHO individuals switch to the MUO phenotype after 4–20 years of follow-up [33, 52–57]. Indeed, it was shown in the North West Adelaide Health Study that MHO is a transient phenotype in about one-third of these individuals, and that those who maintained a MHO phenotype during 5.5–10.3 years of follow-up (i.e. younger individuals with less abdominal adiposity) had a similar T2D and cardiovascular disease risk compared to metabolically healthy, normal weight participants [33]. In the Nurses’ Health Study (median follow-up of 24 years), women with MHO had a higher CVD risk than women with metabolically healthy normal weight, but the risk was considerably higher in women who converted to MUO [50]. In agreement with these observations, it was shown in a community-based population in China, in which ~45% of individuals with MHO developed MUO (follow-up period of 4.4 years), that individuals who experienced transient MHO, but not those with persistent MHO, demonstrated an elevated risk of subclinical atherosclerosis [58]. Population-based studies in Korea (Korean NHIS datasets (2002–2017, mean follow-up of 3.7 years)) and Norway (Nord-Trøndelag Health Study, mean follow-up of ~12 years) indicated that people with persistent MHO during follow-up were, however, not protected against heart failure [59, 60]. Taken together, although MHO seems a transient state in the majority of people, those with persistent MHO have a lower risk of chronic cardiometabolic diseases. One must take into account that the decline in cardiometabolic health associated with aging, the adverse metabolic consequences of prolonged excess adiposity, and the tendency to accumulate fat mass with age will influence the (lack of) stability of the MHO phenotype [41]. The development of IR and elevation of fasting glucose concentration seem major factors related to the transition from MHO to MUO [61]. In line, people with higher BMI, older age, more severe metabolic perturbations, a poor lifestyle index, and body weight gain seem to have a greater risk of transitioning from MHO to MUO [53, 62–67].

## Lifestyle interventions to improve cardiometabolic health and counteract the transition from metabolically healthy to unhealthy obesity

A healthy lifestyle can reduce the risk of cardiometabolic complications and many chronic diseases significantly. Several large population studies have investigated habitual dietary intake in people with MHO and MUO. Most of these studies found that total dietary energy intake and macronutrient composition was similar between individuals with MHO and MUO [35, 68, 69]. In accordance with these observations, no difference in diet quality, appraised as the consumption of Mediterranean-style and DASH-style diets, was found between individuals with MHO and MUO in the US National Health and Nutrition Examination Survey [70]. An intriguing observation was made, indicating that women aged 19–44 years with MHO exhibited a higher total Healthy Eating Index 2005 (HEI-2005) score, which indicates better dietary quality based on the 2005 US National Dietary Guidelines, compared to women with MUO. However, for men aged 19–44 years or adults aged 45–85 years, no significant differences were found in the HEI-2005 total scores between MHO and MUO individuals [71]. Although calorie intake and dietary macronutrient composition did not differ between MHO and MUO individuals in several studies, differences between these metabolic phenotypes were found in number of daily servings of fruit and vegetables, dairy, meats, fats and high fat/sugar food and drinks in some [35, 68, 71–73] but not all [74] studies, with MHO individuals consuming less sugar, sugar-sweetened beverages, and saturated fat and more whole fruits, whole grains, and protein from vegetable sources. Importantly, these results should be interpreted with caution, because of the limitations in the assessment of dietary intake [75, 76] as well as in the definition of MHO in the current studies.

The first treatment recommended for body weight and cardiometabolic health management is lifestyle intervention [77]. To what extent are lifestyle interventions able to prevent the transition from MHO to MUO, or reverse metabolic perturbations in people with MUO? In the Tübingen Lifestyle Intervention Program, achieving ~9 kg median weight loss over 9 months through intensive lifestyle intervention consisting of diet changes and increased physical activity, was associated with improved metabolic health (i.e. conversion from MUO to a healthier metabotype [78]. Interestingly, BMI and liver fat content were independent predictors of the metabolic improvements in the latter study [78].

Would it also be possible to improve cardiometabolic health in MUO with nutritional or lifestyle interventions in the absence of marked weight loss? A very recent study investigated whether adherence to healthy diets was related to the incidence of metabolically unhealthy phenotypes in adults in various BMI categories. It was shown that high compliance with the Dietary Approaches to Stop Hypertension (DASH), Mediterranean (MeDi), and Mediterranean-DASH intervention for neurodegenerative delay (MIND) diets was related to a reduced risk of metabolically unhealthy normal weight. Furthermore, adherence to these dietary patterns was negatively associated with the incidence of MUO in those with MHO at baseline [79]. Furthermore the large Prevención con Dieta Mediterránea (PREDIMED) trial demonstrated that the Mediterranean diet lowered the risk of cardiovascular events by about 30% in comparison to the control diet, without large effects on body weight [80]. Interestingly, findings from the PREDIMED study also demonstrated that even without substantial weight loss, adherence to a Mediterranean diet can promote the transition from MUO to a healthy metabotype, and protects against worsening of metabolic health in MHO [81]. Comparable findings were seen in several other dietary intervention studies [82].

Differences in body composition, tissue-specific metabolism and insulin sensitivity between men and women not only underlies sexual dimorphism in cardiometabolic disease risk, but may also affect the response to prevention and treatment strategies in a sex-specific manner [22]. Indeed, dietary interventions have a differential effect on body weight, weight maintenance and cardiometabolic risk factors in men compared to women, as extensively reviewed elsewhere [23]. The recent PREVIEW study investigated age- and sex-specific effects of a low-energy diet (LED), followed by a 3 year lifestyle-based weight maintenance intervention in overweight adults with prediabetes [83]. A lifestyle intervention had less beneficial effects with respect to body composition and cardiometabolic health markers in older than younger adults, despite better weight maintenance, which might be due to the loss of more fat-free mass in older adults. The LED, followed by a lifestyle intervention, showed less beneficial effects on body weight and body composition in women than men [83]. The available evidence suggests that factors such as age and sex should be taken into account to optimize prevention and treatment strategies for those at risk of or already living with cardiometabolic diseases.

## Tissue insulin resistance metabotype: the concept

Insulin plays a crucial role in regulating nutrient partitioning within the body. Insulin resistance (IR) encompasses impaired insulin action in various tissues, including muscle, liver, adipose tissue, gut, and brain. This dysfunction may occur before the onset of cardiometabolic diseases.

Importantly, IR may develop in different organs but the severity may vary between organs. Indeed, in individuals with prediabetes, the state of impaired glucose tolerance (IGT) or the state of impaired fasting glucose (IFG) and are characterized, among other factors by more pronounced peripheral (skeletal muscle) or more pronounced liver IR [26]. Consistent with this, our previous research demonstrated that individuals with impaired fasting glucose (IFG) did not exhibit disruptions in skeletal muscle lipid turnover, whilst in individuals with impaired glucose tolerance (IGT), there were disturbances in skeletal muscle lipid handling. These disturbances were accompanied by impaired postprandial insulin sensitivity, an increase in postprandial triglyceride (TAG) extraction, and a reduction in muscle lipid turnover [84].

Individuals with more pronounced liver insulin resistance (LIR) and individuals with more pronounced muscle insulin resistance (MIR) can already be distinguished in the overweight or obese state [85]. Using a 5 or 7-points oral glucose tolerance test, wherein insulin and glucose concentrations were measured, we calculated hepatic insulin resistance index (HIRI) and muscle insulin sensitivity index (MISI). These indices were validated against a hyperinsulinemic clamp, a well-established method for assessing insulin sensitivity [86]. Additionally, we optimized the MISI calculation by means of cubic splining [87]. Individuals with LIR have a distinct metabolome [88] and lipidome profile [89] as compared to individuals with more pronounced MIR. Furthermore, an enriched inflammatory gene expression profile was particularly present in abdominal subcutaneous adipose tissue of individuals with primarily MIR [90]. Additionally, an altered extracellular matrix remodelling gene expression profile was present in individuals with pronounced LIR [90]. In line with the former findings, in two population-based cohorts, the Cohort on Diabetes and Atherosclerosis Maastricht and The Maastricht Study, we observed that an elevated systemic low-grade inflammation profile, as indicated by the combined score of plasma markers associated with low-grade inflammation, showed a specific association with MIR but not with LIR [90]. The connection between adipose tissue inflammation and IR has been previously shown, but our data further expand on these findings by demonstrating the tissue-specific nature of this relationship. Nevertheless, only one-third of the population with overweight harbor the MIR/LIR phenotype, and it is evident that more tissue metabotypes can be identified (hypothesized around six assuming an even distribution). Recent findings show that adipose tissue IR and whole-body IR, as reflected by Homeostatic Model Assessment of Insulin Resistance (HOMA-IR), not always coincide [91]. Adults that are discordant for adipose tissue insulin resistance and HOMA-IR had unique features related to visceral fat, plasma triglycerides and basal metabolic rate [91].

Furthermore, tissue metabotypes may also vary based on tissue fat accumulation and may vary between sexes. Body composition profiles, as assessed by whole body magnetic resonance imaging (MRI), may further our understanding of the complex interplay between muscle and liver metabolism and ectopic fat and adipose tissue fat accumulation. Indeed, distinct etiologies towards cardiometabolic health outcomes have been shown for discordant visceral and liver fat phenotypes [92] as well as discordant liver and muscle fat/mass phenotypes [93]. Finally, sex-specific differences in the accumulation of surplus lipids, mobilization of stored lipids, as well as substrate supply and utilization in critical metabolic organs (such as skeletal muscle, adipose tissue, and the liver) are linked to variations in tissue-specific insulin sensitivity and cardiometabolic risk profiles between men and women (Fig. 1). Premenopausal women have, in general, an increased liver and muscle insulin sensitivity as compared to males [23] (Fig. 1). We previously showed that in women, but not in men, LIR was positively associated with the sum of plasma diacylglycerols and triacylglycerols (TAG) [89]. The latter results remained consistent even after adjusting for body composition and body fat distribution, suggesting that factors beyond body composition play a significant role in these sex-specific differences. Furthermore, these women had lower plasma TAG and higher HDL concentrations and a reduced LIR as compared to men. In general, healthy premenopausal women appear to possess a greater capacity for fat storage without incurring detrimental cardiometabolic health risks, a phenomenon often referred to as the female advantage [23]. Our findings revealing a deterioration in blood lipid profile among women as LIR progresses are particularly noteworthy. These findings imply that women with LIR eventually "catch-up" with men in terms of CVD risk highlighting a sex-specific relationship between (L)IR and cardiometabolic risk. This is consistent with findings of Kim and Reaven [94], who showed that the female advantage is not solely explained by differences in insulin action itself. Instead, they found that the female advantage arises from an attenuation of the association between IR and CVD risk, particularly evident in younger individuals (aged below 51 years). The mechanisms behind these intruiging sex-specific metabolic differences remain to be determined. In summary, distinct tissue metabotypes can be identified in individuals with overweight and obesity which may represent different etiologies towards cardiometabolic diseases.

## Precision nutrition based on tissue-IR metabotype

Post-hoc analyses in large intervention studies show responders and non-responders that feed back to tissue metabotype [5–8, 83]. Parameters associated with glucose metabolism and IR, including plasma glucose and insulin concentrations and derived indices can serve as valuable predictors of the outcome of a dietary intervention [5, 6, 11]. Post-hoc evidence from an analysis of the CORDIO-PREV-DIAB study shows an interaction between dietary macronutrient composition and tissue metabotype [8]. In the latter study, researchers compared the effects of a Mediterranean diet, rich in olive oil, to a low-fat high complex carbohydrate diet in relation to outcomes of glucose metabolism. After the study, a post hoc analysis was performed, dividing participants based on their baseline tissue-IR phenotype. The results indicated that individuals with LIR may derive greater benefits from the low-fat high complex carbohydrate diet, displaying a more pronounced increase in disposition index (a composite marker considering insulin secretion adjusted for insulin sensitivity) as compared to the MIR phenotype. On the other hand, individuals with the MIR phenotype appeared to benefit more from the Mediterranean diet, showing a higher increase in the disposition index compared to those with the LIR phenotype.

**Fig. 2:**
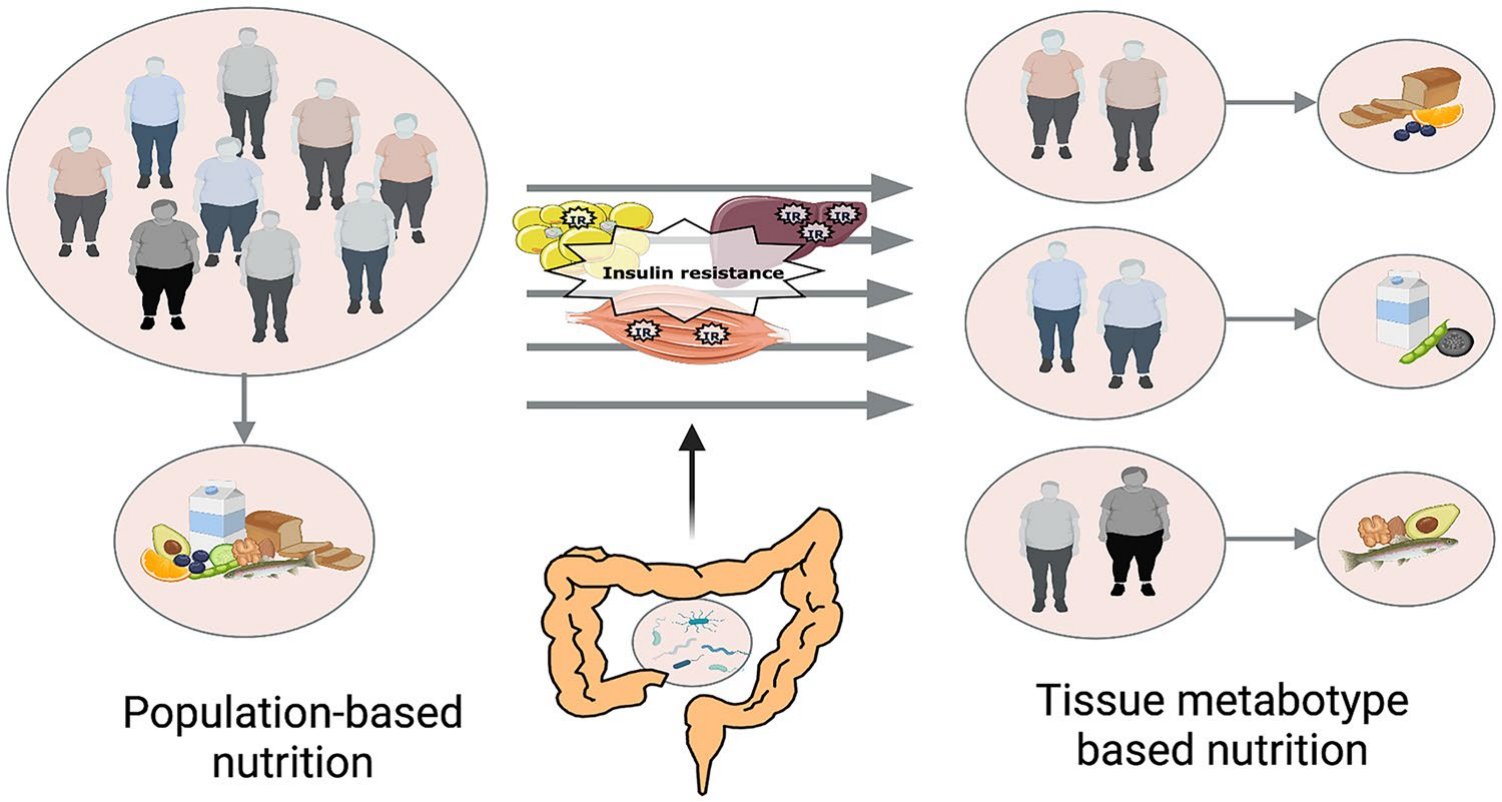
The depicted precision nutrition concept is based on the definition of tissue metabotypes related to parameters of tissue-specific metabolism like MIR, LIR and adipose IR, tissue fat accumulation and microbial composition; this strategy will improve insulin sensitivity, blood glucose homeostasis and cardiometabolic risk compared to current, one-size-fits-all dietary guidelines in the population with overweight. Created with BioRender.com

We recently provided the proof-of-concept that a precision nutrition strategy according to an individual’s tissue metabotype, within the context of healthy dietary guidelines, results in a clinically relevant further improvement in insulin sensitivity and cardiometabolic health (C-reactive protein and plasma triacylglycerol concentrations) in individuals with overweight or obesity, independent of body weight change [14]. Individuals with the LIR phenotype responded better to a diet high in mono-unsaturated fatty acids, whilst individuals with the MIR phenotype responded better to a diet low in fat and high in protein and fiber. Our data show the potential of precision nutrition based on tissue metabotypes. The latter precision nutrition concept is depicted in Fig. 2. Thus, while a diet based on existing dietary guidelines may promote general health for many individuals, it is becoming increasingly evident that precision or subgroup-based dietary approaches might be necessary for optimal dietary prevention or treatment outcomes.

## Precision nutrition based on microbial phenotypes

During recent years, research has shown that a disbalance in gut microbiota communities and functionality is implicated in a large number of chronic metabolic diseases including obesity, IR, T2D and cardiometabolic risk [95]. Early studies reported an increased Firmicutes-to-Bacteroidetes ratio in both rodents and humans with obesity in comparison to lean individuals [96, 97]. However, subsequent studies have presented conflicting findings, with some reporting no significant difference in the Firmicutes-to-Bacteroidetes ratio between lean individuals and individuals with obesity, and others even showing a decreased ratio [98, 99]. Consistent findings from various studies have revealed that overweight, moderate obesity, IR, and T2D are associated with decreased microbial richness and diversity compared to lean, healthy individuals [100]. In-depth characterization of people with overweight or obesity has demonstrated that microbial gene richness is negatively associated with various metabolic parameters, including fat mass, leptin levels, fasting insulin, HOMA-IR, systemic inflammation, and TAG levels [100]. Moreover, low microbial gene richness is prevalent in severe obesity, with approximately 75% of individuals affected, while the numbers range from 23 to 40% in lean individuals or those with overweight or moderate obesity [100, 101]. Dietary interventions have been shown to improve clinical phenotypes in individuals with low microbial gene richness. However, these interventions appear less effective in improving inflammatory markers in this subgroup. As a result, low gene richness may serve as a predictive factor for the efficacy of interventions [101].

Our gut microbiota produces a large variety of health-modulating products, including short-chain fatty acids (SCFA) and branched-chain fatty acids (BCFA), by fermenting indigestible food components. It is increasingly clear that these products are essential for host health [95, 102, 103]. The major SCFA are acetate, propionate and butyrate. SCFA are produced by saccharolytic fermentation mainly in the proximal and transverse colon, with beneficial effects on metabolic health, whereas BCFA produced by proteolytic fermentation in the distal colon have general adverse effects on host health (as reviewed in 105 and 106). Interestingly, the gut microbiome of IR individuals has been shown to have an increased biosynthesis potential and decreased uptake and catabolism of branched chain amino acids (BCAAs, largely driven by *Prevotella copri and B. vulgatus*), which have been linked to adverse metabolic effects [104]. Additionally, metabolically compromised individuals as well as patients with T2D have an altered microbial functionality and a decreased fermentation capacity when compared with healthy individuals, characterised in particular by a lower abundance of butyrate producing bacteria [105–107]. Previous research conducted by our group revealed that acutely administering acetate directly to the distal colon led to increased levels of circulating acetate in males with overweight. This intervention resulted in elevated concentrations of the satiety-stimulating hormone peptide YY and reduced levels of the cytokine TNF-α. Notably, the acetate administration also resulted in a significant increase (25%) in fasting fat oxidation. Contrary, when acetate was administered in the proximal colon, no significant effects on the metabolic profile were observed. This suggests that the specific location of acetate administration in the colon plays a crucial role in its metabolic effects [108]. Thus, increasing the formation of SCFA in the distal colon by enhancing dietary fiber availability could be a critical factor in improving metabolic health. In particular, since a higher distal carbohydrate fermentation may help reduce detrimental proteolytic fermentation. This concept of ‘microbial substrate switch’ emphasizes the importance of dietary strategies that promote the growth and activity of beneficial gut microbes in the distal colon and might provide a novel dietary strategy for preventing and/or treating metabolic diseases (as reviewed in 105).

Interestingly, the tissue IR metabotypes, MIR and LIR are characterized by a differential microbial composition with the LIR metabotype having a higher abundance of SCFA producing genera [109]. In line, modification of microbial composition by either fecal transplant [105, 110] or by dietary maize fiber intervention [111] has been shown to affect peripheral insulin sensitivity more than LIR. Additionally, it was recently shown that microbial composition of individuals with IFG, characterized by LIR, resembles more the normal glucose tolerant state, whilst individuals with IGT, characterized by MIR, show a microbial dysbiosis resembling the T2D state with a reduced abundance of butyrate producing bacteria [107]. Combined, variation in microbial composition and functionality (fermentation) may affect (dys)metabolism in a tissue-specific manner.

Inter-individual differences in gut microbiota composition and functionality may be linked to an altered responsiveness to (dietary) interventions. Initial microbial phenotype has been shown to predict intervention outcome after dietary fiber interventions [112], after feces transplantation [110], bariatric surgery [113] or ingestion of non-caloric sweeteners [114]. Microbial responses to fiber specific interventions have also revealed responders and non-responder phenotypes related to the magnitude of production of the fiber derived SCFA [115]. We recently showed that persons living with overweight or obesity and prediabetes show changes lower postprandial insulin sensitivity in response to short-term administration of the prebiotic fiber long-chain inulin (combined with resistant starch) compared to lean individuals [116], along with reduced plasma concentrations of the SCFA butyrate. These effects were fiber-specific i.e. were not seen when administering beta-glucan and resistant starch, indicating complex structure–function relationships of dietary fibers [116]. These data suggest that the degree of saccharolytic fermentation and related SCFA production may be an important determinant of intervention outcome. Additionally, our data show a lack of response in individuals with prediabetes, which is consistent with the observation that a 4-week oral administration of butyrate altered metabolism and insulin sensitivity in lean individuals but not in individuals with obesity and IR [117]*.* Combined, the initial microbial composition and related SCFA production may be important determinants of dietary intervention outcome.

## Future perspectives

By investigating the relationship between metabotypes and intervention outcomes, we can identify which dietary approaches are most suitable for specific individuals or subgroups at risk for chronic cardiometabolic diseases. A better understanding of the biological, psychological and socio-economic factors that may underlie the MHO and MUO phenotypes will generate important knowledge on obesity-related cardiometabolic chronic diseases that may aid in the development of more personalized interventions. Based on the limited resources that are available for lifestyle interventions, it may be reasonable to prioritize interventions to people with MUO to improve the cost-effectiveness of interventions. A study investigating the effect of phentermine/topiramate-induced weight loss on the prevention of T2D in subjects who were stratified by the Cardiometabolic Disease Staging score (very similar to the MHO/MUO concept) in those with a high or a low cardiometabolic risk provided support for this assumption [118], demonstrating that numbers needed to prevent one case of T2D over about 1 year were 120 in the low-risk group but 24 in the high-risk group [118]. Additionally, research has shown that nutritional or lifestyle interventions can significantly improve cardiometabolic health, even in the absence of substantial weight loss. This finding indicates that the focus should be on promoting a healthy lifestyle rather than focusing on weight loss as the primary goal. In this respect, a diet based on existing guidelines for healthy nutrition, which emphasizes whole foods, a variety of nutrients, and moderation in portion sizes, can indeed serve as a good foundation for promoting health in the general population. Nevertheless, these guidelines may not represent the optimal diet for all. As evidenced by the recent proof of concept in the PERSON study [14], dietary macronutrient modulation according to tissue IR metabotype within the context of healthy dietary guidelines may further improve cardiometabolic health. Thus, precision nutrition based on tissue metabotype may be more effective in cardiometabolic disease prevention as compared to general dietary guidelines. These data demonstrate that the different metabotypes towards T2D and cardiometabolic diseases have to be considered and may lead to more effective nutritional or lifestyle prevention and treatment strategies. In this consideration, age and sex-related differences in tissue metabotypes and related microbial composition and functionality, as important drivers or mediators of dietary intervention response, have to be taken into account. Overall, investing in more prospective trials focused on precision nutrition will contribute significantly to advancing the field and optimizing dietary prevention for individuals at risk for chronic cardiometabolic diseases.

## Data Availability

N/A.

## Abbreviation

BMI: Body Mass Index
BCFA: Branched-Chain Fatty Acids
CVD: CardioVascular Diseases
DASH: Dietary Approaches to Stop Hypertension
HIRI: Hepatic Insulin Resistance Index
HOMA-IR: Homeostatic Model Assessment of Insulin Resistance
IFG: Impaired Fasting Glucose
IGT: Impaired Glucose Tolerance
IR: Insulin Resistance
LIR: Liver Insulin Resistance
LED: Low-Energy Diet
MeDi: Mediterranean
MRI: Magnetic Resonance Imaging
MIND: Mediterranean-DASH intervention for Neurodegenerative Delay
MHO: Metabolically Healthy Obesity
MUO: Metabolically Unhealthy Obesity
metabotypes: Metabolic phenotypes
MIR: Muscle Insulin Resistance
MISI: Muscle Insulin Sensitivity Index
PREDIMED: Prevención con Dieta Mediterránea
SCFA: Short-Chain Fatty Acids
TAG: Triacylglycerols
T2D: Type 2 Diabetes

## Code availability

N/A.
